# Antigenic Diversity of Enteroviruses Associated with Nonpolio Acute Flaccid Paralysis, India, 2007–2009

**DOI:** 10.3201/eid1811.111457

**Published:** 2012-11

**Authors:** C. Durga Rao, Prasanna Yergolkar, K. Subbanna Shankarappa

**Affiliations:** Author affiliations: Indian Institute of Science, Bangalore, India (C.D. Rao, K.S. Shankarappa);; National Institute of Virology–World Health Organization–South East Asia Region Polio Laboratory Network, Victoria Hospital, Bangalore (P. Yergolkar);; National Institute of Virology, Bangalore (P. Yergolkar);; Victoria Hospital, Bangalore (P. Yergolkar)

**Keywords:** Poliomyelitis, polio acute flaccid paralysis, nonpolio acute flaccid paralysis, nonpolio enteroviruses, enterovirus 71, coxsackievirus, echovirus, poliovirus, viruses, India

## Abstract

Because of the broadened acute flacid paralysis (AFP) definition and enhanced surveillance, many nonpolio AFP (NP-AFP) cases have been reported in India since 2005. To determine the spectrum of nonpolio enterovirus (NPEV) serotypes associated with NP-AFP from polio-endemic and -free regions, we studied antigenic diversity of AFP-associated NPEVs. Of fecal specimens from 2,786 children with NP-AFP in 1 polio-endemic and 2 polio-free states, 823 (29.5%) were positive for NPEVs in RD cells, of which 532 (64.6%) were positive by viral protein 1 reverse transcription PCR. We identified 66 serotypes among 581 isolates, with enterovirus 71 most frequently (8.43%) detected, followed by enterovirus 13 (7.1%) and coxsackievirus B5 (5.0%). Most strains within a serotype represented new genogropups or subgenogroups. Agents for ≈35.0% and 70.0% of culture-positive and -negative cases, respectively, need to be identified. Association of human enterovirus with NP-AFP requires better assessment and understanding of health risks of NPEV infections after polio elimination.

## Abstract 2

Three new enterovirus 71 genogroups are geographically widely disbursed.

Acute flaccid paralysis (AFP) is defined as sudden onset of weakness and floppiness in any part of the body in a child <15 years of age or paralysis in a person of any age in whom polio is suspected (*1*). It is a complex and broad clinical syndrome associated with a wide range of microbial and nonmicrobial agents and immune processes; clinical presentations and numbers are influenced by environmental and geographic factors. To cast a wider net for poliovirus detection and to maximize sensitivity so that every poliomyelitis case is detected, in 2005, the Global Poliomyelitis Eradication Initiative adapted AFP as a surveillance tool and broadened the case definition of AFP in India. The expanded case definition of AFP encompasses causes of nonpolio AFP (NP-AFP), including Guillian-Barré syndrome, transverse myelitis and traumatic neuritis, and ambiguous cases (*1*).

With the launch of the Global Poliomyelitis Eradication Initiative in 1988 for effective vaccination, surveillance, and monitoring of wild poliovirus transmission toward the target of polio eradication, the number of wild polio AFP cases declined remarkably from ≈300,000 to 974 in 2010 globally (*2*). Introduction of Pulse Polio Immunization, in addition to routine administration of oral polio vaccine, effectively interrupted indigenous wild poliovirus transmission and led to a remarkable decline in the number of poliomyelitis cases from ≈35,000 cases annually during 1994–1995 to 66 in 2005 in India. However, during 2006–2009, the number of polio cases hovered at ≈559–874 each year (*2*–*5*), with most cases reported primarily from the 2 northern states of Uttar Pradesh and Bihar, in which wild poliovirus remained endemic. The last case of type 2 wild poliovirus globally occurred in 1999 in India. Introduction of bivalent oral polio vaccine types 1 and 3 resulted in a dramatic decline in wild poliovirus cases to 42 in 2010 and only 1 case reported in January 2011 (*2**–**5*). India is now considered a polio-free nation by the Government of India, National Polio Surveillance Project (NPSP) and by the World Health Organization (WHO)/South-East Asia Regional Office and WHO.

However, analysis of WHO-monitored polio surveillance data on the number of AFP, polio AFP, and NP-AFP cases available at the public domains (www.polioeradication.org/; www.searo.who.int/vaccine; www.npspindia.org/) (*2*–*5*) from 1998 through June 2, 2012, in India shows that concomitant with the phenomenal elimination of wild poliovirus transmission in India was an annual increase in the number of reported AFP cases from 2005 to date throughout the country (*2*–*4*). Although 8,103–9,705 were reported during 1998–2003, a total of 55,782 and 60,883 cases were reported during 2010 and 2011, respectively. Through June 2, a total of 20,677 AFP cases were reported in India during 2012, compared with 18,625 during the corresponding period in 2011 (*4*). This large increase in NP-AFP cases, which represent AFP cases caused by agents other than poliovirus, probably reflects the excellent implementation of the expanded definition of AFP and highly sensitive surveillance and detection methods used by NPSP in India from 2005 onwards, in contrast to the other polio-endemic countries, i.e., Pakistan, Nigeria, and Afghanistan, where the expanded AFP surveillance is not in place (*1*–*5*). The large increase in the NP-AFP rate from 1.45 and 1.97 per 100,000 children during 1998–2003 to 16.20 in 2011 (*3**–**5*) further reflects the excellent operational performance of the expanded AFP surveillance in India.

The genus *Enterovirus* within the family *Picornaviridae* comprises a diverse group of 10 species, of which 7 are associated with a wide spectrum of acute and chronic human diseases (*6*,*7*). Human enteroviruses (HEVs) are ubiquitous, infecting ≈1 billion persons worldwide. Although the actual incidence of enteroviral diseases is not known, most infections are thought to be asymptomatic, with ≈1% resulting in severe illness with high rates of death, especially in infants and young children (*6*). The >100 HEV serotypes comprising echoviruses (E), coxsackieviruses A (CAV) and B (CBV), polioviruses, and newer enteroviruses (EV) have been grouped into 4 species—HEV-A, HEV-B, HEV-C, and HEV-D—with poliovirus being part of HEV-C. Recently, rhinoviruses also have been included in the genus *Enterovirus* (*8*).

Molecular typing methods based on reverse transcription PCR (RT-PCR) amplification, nucleotide sequencing of the complete or the 3′ portion of the viral protein (VP) 1 gene, and comparison of the derived sequences with those of prototype and variant HEVs in the databases are widely used to identify EV types in clinical samples (*9*,*10*). In the current most commonly used molecular typing scheme, homotypic viruses generally share at least 75% nt identity and 85%–88% aa identity in VP1 (*9*,*10*).

Although nonpolio enteroviruses (NPEVs) are a major cause of AFP (*6**,**7**,**11*) and NP-AFP cases are being detected in large numbers, detailed knowledge is lacking about the serotypes associated with NP-AFP or other enteroviral diseases in India. We aimed to determine the spectrum of NPEV serotypes associated with NP-AFP from polio-endemic and polio-free regions of India with a view to develop strategies against the so-far unrecognized viral infections.

## Materials and Methods

### AFP Samples and Processing Laboratory

The National Institute of Virology Bangalore Unit in Victoria Hospital (Bangalore, India) is a WHO-accredited National Polio Laboratory for receiving, processing, and analyzing AFP specimens for polio and NPEVs (*5*,*12*). At least 2 specimens, obtained ≈24 hr apart, from each person with AFP were collected during 2007–2009. The National Institute of Virology–National Polio Laboratory receives fecal specimens of persons with AFP in accordance with WHO NPSP guidelines from the southern states of Karnataka and Kerala, 5 districts of the polio-endemic Uttar Pradesh State (Pilibhit, Badaun, Bareilly, Rampur, and Shahjahanpur), and other districts as and when required by the NPSP.

The chloroform-treated supernatants from 2,786 first-collection fecal samples from NP-AFP patients and second specimens from 310 of the patients were examined for EVs by observing cytopathic effects in L20B and RD cells. Poliovirus-positive isolates were further identified by neutralization tests, RT-PCR and ELISA following WHO-prescribed protocols (*12*) and sequence analysis of VP1 gene for determining vaccine-derived polioviruses. RT-PCRs have replaced all the earlier methods for identifying wild and vaccine polioviruses. All fecal specimens are stored for 1 year at −20°C and discarded by autoclaving. The virus-positive cell culture supernatants were stored at −20°C until completion of reporting, and only NPEVs were retained for further studies. RNA was extracted from 1,129 samples from children with NP- AFP, which include 823 NPEV-positive individual cases and 138 NPEV-positive second specimens. A total of 174 NPEV-negative fecal samples, including 16 second specimens, were used as controls to determine whether any of the samples negative for EV in cell culture became positive in RT-PCR.

### Healthy Children

Fecal specimens from 780 apparently healthy children 3 months–3 years of age who did not receive vaccine and did not have diarrhea or any other illnesses during the 2 weeks before sample collection from different localities in the Bangalore community were used as healthy controls to determine the frequency of EV detection in healthy children. We obtained the necessary approvals from Institutional Biosafety and Ethical committees for carrying out the work.

### Laboratory Analyses

Total RNA was extracted from 200 µL of NPEV–positive RD cell culture supernatants or clarified chloroform extracted fecal samples by using RNeasy mini kit (QIAGEN, Hilden, Germany). RNA was eluted in 100 µL of RNase-free water and stored at −80°C. EV species–specific degenerate primers were constituted into 4 sets (Technical Appendix). Set 1 contained EV-B and EV-C–specific primers; set 2 contained primers specific for EV-A, EV71, and EV-D and sets 3 and 4 consisted of primers specific for Aichi virus/kobuvirus and klasseviruses and for cardioviruses and human cosaviruses, respectively. VP1 region was amplified by 1-step RT-PCR (QIAGEN) with reverse transcription at 40–42°C for 45 min depending on the primer, followed by heating at 94°C for 15 min. The first 2 PCR cycles were performed with melting, annealing, and extension at 94°C (40 s), 45–50°C (30 s), and 68°C (2 min), respectively. PCR was continued for 40 cycles with annealing and extension at 55°C (30 s) and 72°C (2 min), respectively. VP1 PCR fragments were sequenced (Macrogen Inc., Seoul, South Korea; SciGenom, Cochin, India) either directly by using primers corresponding to arbitrary sequences present at the 5′ end of the PCR primers or by using M13 forward and reverse primers after cloning the inserts between *Eco*RI or *Bam*HI and *Hin*dIII sites in pBluescript KS^+^ (Agilent Technologies, Santa Clara, CA, USA).

### Phylogenetic Analyses

EV serotypes were identified by comparing VP1 nucleotide and deduced amino acid sequences from NP-AFP isolates among themselves and with those of other prototype or variant strains belonging to all known serotypes available in GenBank. Phylogenetic analyses were conducted by using MEGA5 (*13*) as described in the legends to the figures. GenBank accession numbers of reference strains used for sequence comparisons are available at www.pcornastudygroup.com, some of which are indicated in the trees. The VP1 gene GenBank accession numbers of 618 India NP-AFP isolates are HQ454497–454499 and JN203499–JN204113.

## Results

### Frequency of NPEV Detection in Children with NP-AFP and in Healthy Children

A total of 2,786 fecal specimens from individual NP-AFP patients and second-collection specimens from 310 of the patients were examined for EV. Samples from 823 (29.5%) NP-AFP patients were positive for virus growth in RD cells. The percentage of virus positivity varied from 24.0% to 33.0% in different years from the 3 states. The remaining ≈70% samples were negative for EV in RD cells (Table). Of the 823 NPEV-positive cases, 532 (64.6%) yielded VP1-specific PCR fragments corresponding to EV-A, EV-B, or EV-C species. Sequencing of VP1 from 154 second specimens confirmed the serotype specificity of the isolate determined from the first sample (Tables 1 and 2 in Technical Appendix). Whereas the species-specific primers yielded a PCR product of ≈1,200 bp, the EV71–specific primers F1 and R1 yielded an 850-bp fragment. VP1 region from 291 (35.4%) samples could not be amplified by using primers designed for EV-A, EV-B, EV-C, and EV-D, bovine enterovirus, porcine enterovirus, klassevirus, kobuvirus, human cosavirus, or parechovirus under various PCR conditions (*8*,*14*–*20*). Four (2.3%) of 174 NPEV-negative samples showed positivity in RT-PCR (Table; Table 1 in Technical Appendix).

**Table:** Analysis of fecal samples in 2,786 children with AFP and in 780 healthy children for virus growth in RD cells and for viral protein 1 gene RT-PCR, India, 2007–2009*

State/RD cell status	Total no. AFP samples (second samples)	No. samples used for RNA extraction			Total no. (%) RD-positive patients	No. (%) RT-PCR-positive patients/RD-positive or -negative patients
		2007	2008	2009		
Karnataka	929 (253)					
Positive	NA	51	75	87	213/676 (31.51)	141/213 (66.2)
Negative	NA	48	60	32	0	4/158 (2.5)†
Healthy children	NA	240	250	290	20/780 (2.56)†	18/20 (90.0)†
Uttar Pradesh: positive	1,833 (39)	129	165	232	526/1,794 (29.32)	331/526 (62.93)
Kerala	334 (18)					
Positive	NA	22	45	17	84/316 (26.6)	60/84 (71.7)
Negative	NA			18		
Total	3,096	490	595	676	823/2,786 (29.54)	532/823 (64.64)

*AFP, acute flacid paralysis; RT-PCR, reverse transcription PCR; NA, not applicable. †Not included in RD cell–positive or RT-PCR–positive samples from children with AFP.

Of the 780 fecal specimens from apparently healthy children, ≈20 (2.6%) fecal samples were positive for NPEV in cell culture, and ≈23 (3.0%) were RT-PCR-positive with the same primers used for AFP samples (Table). Although only 4 (0.9%) of 450 samples collected during winter months (November–March) were positive for NPEV, most virus-positive samples (16 [4.8%] of 330) were those collected in other months, suggesting seasonal incidence of NPEV infections in Bangalore.

### Extreme Antigenic Diversity of EVs Associated with NP-AFP

**Figure 1:**
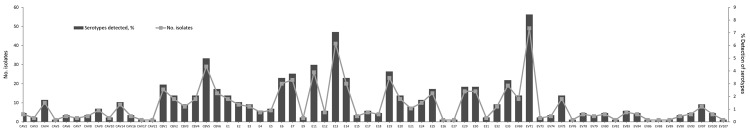
Strains belonging to each serotype detected in children with acute febrile paralysis, India, 2007–2009. CA, coxsackievirus; E, echovirus; EV, enterovirus.

**Figure 2:**
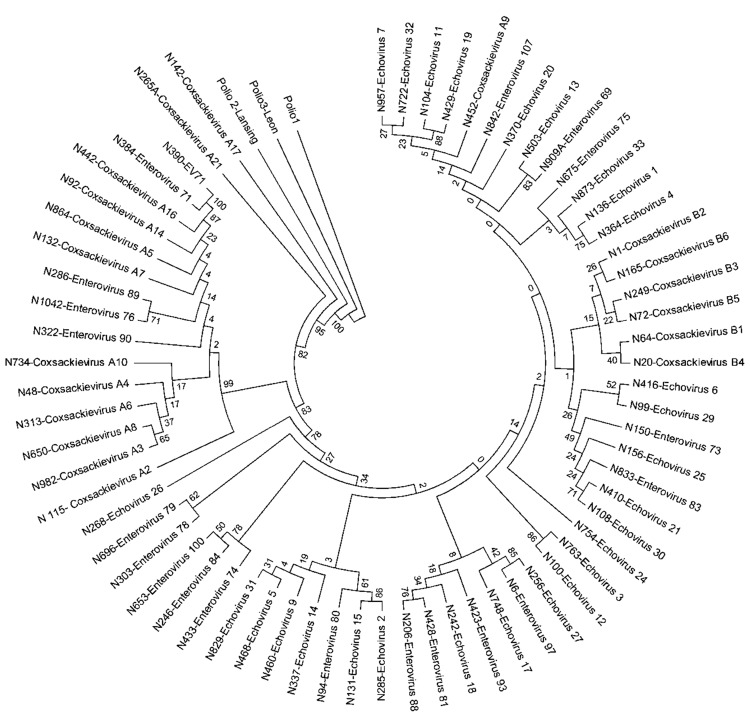
Phylogenetic tree based on viral protein 1 gene nucleotide sequences of strains representing each of the 66 enterovirus serotypes detected in children with acute febrile paralysis, India, 2007–2009. The phylogenetic tree was constructed by using the maximum-parsimony method and with search level 1 close-neighbor-interchange algorithm. The percentage of replicate trees in which the associated types clustered together in the bootstrap test (500 replicates) is shown next to the branches. Evolutionary analyses were conducted in MEGA5 (*13*). The viral protein 1 gene sequences of the 3 poliovirus prototypes were used as reference. The strain numbers with their serotype association are indicated. The GenBank accession numbers of VP1 gene for 618 acute febrile paralysis isolates are HQ454497–454499 and JN203499–JN204113.

Comparison of the complete or partial VP1 gene sequences of the clinical isolates with those of prototype and other EVs available in GenBank and phylogenetic analyses (*13*) showed 66 serotypes among isolates associated with NP-AFP (Figures 1, 2). Among the 66 serotypes, EV71 was more frequently detected than others, representing ≈8.4% of the characterized isolates, followed by E13 (7.1%) and CBV5 (5.0%). Although strains belonging to 6 serotypes (E6, E7, E11, E14, E19, and E33) each accounted for 3.3%–4.5% of the characterized strains, those belonging to 13 serotypes (CVA4, CBV1, CBV2, CBV4, CBV6, E1, E20, E24, E25, E29, E30, EV69, and EV75) each represented 1.7%–2.9%. The frequency of detection of each of the other serotypes ranged from 0.2% to 1.6% (Figure 1; Tables 1 and 2 in Technical Appendix). Only 15 serotypes (CAV4, CBV2, CBV4, E6, E7, E13, E14, E17, E19, E25, E30, E33, EV71, EV75, EV80) were detected in all 3 states. Forty-eight serotypes, including CBV5, were not detected in Kerala, probably because of the relatively small number of samples available from that state or noncirculation of these serotypes. Strains representing 16 serotypes were each detected only 1× or 2× during the period and represented <0.2%–0.3% of the characterized isolates (Figure 1). Strains belonging to EV-C species, to which poliovirus belongs, were rarely observed, with 1 CAV17 strain in Karnataka and 1 CAV21 strain in Uttar Pradesh being detected in samples collected during 2008. Of the 4 NPEV-negative samples that showed positivity in RT-PCR, 3 belonged to CAV4 and the other to CAV8.

Of the 532 NPEV-positive cases, 41 (7.7%) were identified as exhibiting mixed infection by >1 strain, as indicated by RT-PCR and sequence analysis of the cloned fragments. Although 37 cases of mixed infections involved 2 different serotypes, infections involving 3 different serotypes were detected in 4 cases. Although 32 serotypes were detected in mixed infections, CBV4, CBV5, E6, EV69, and EV71 accounted for 12.2%, 12.2%, 14.6%, 17.1%, and 31.7% of these infections, respectively. Mixed infections involving EV69 and CBV4 accounted for 58.3% and 41.7%, respectively, of their total detections (Table 3 in Technical Appendix).

A notable observation was detection of EV71 as the single most prevalent serotype; it accounted for ≈8.5% of the characterized isolates. Although EV71 was undetectable in 2007 and only 1 isolate was detected during 2008 in Karnataka, it was the most frequently detected serotype in Kerala during these 2 years. EV71 appears to have spread from Kerala to the neighboring Karnataka, resulting in its frequent detection in 2009. EV71 was frequently detected in Uttar Pradesh only during 2008 and 2009. Although 26.5% of total EV71 detections involved mixed infection, it alone accounted for 31.7% of mixed infections (Table 3 in Technical Appendix).

### Genetic Relatedness of EV71, E13, and CBV Strains in India with Strains from Other Countries

**Figure 3:**
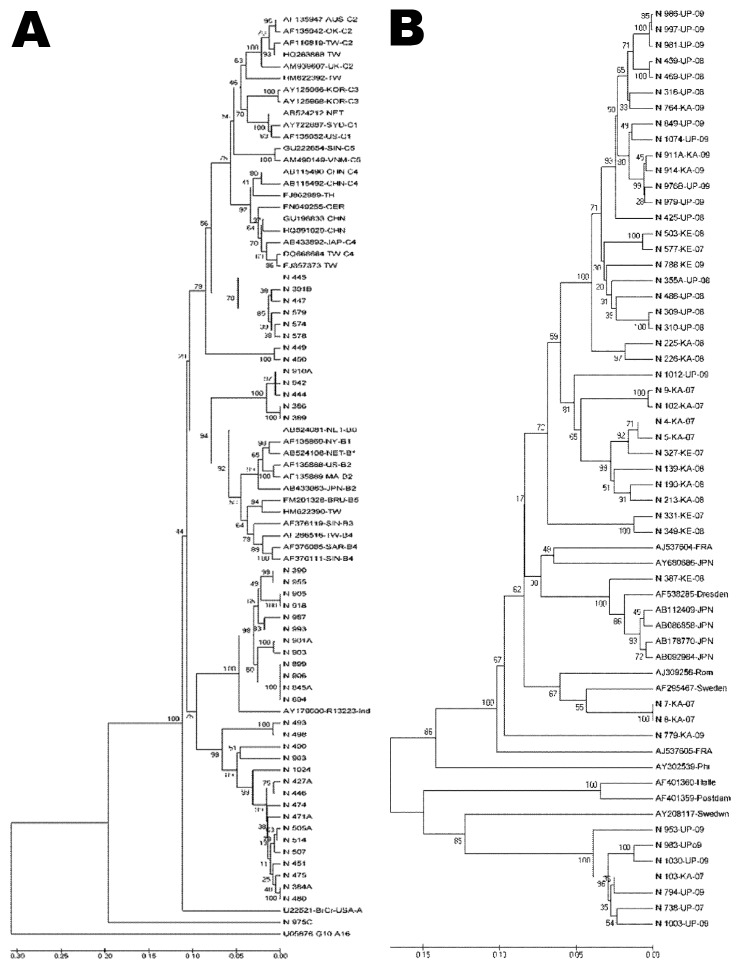
Phylogenetic analyses of viral protein 1 sequences of enterovirus 71 and echovirus 13 strains with those of reference strains representing different genogroups and subgenogroups within a serotype, India, 2007–2009. Multiple sequence alignments were performed by using ClustalW program (www.genome.jp/tools/clustalw/) and phylogenetic analysis by MEGA5 program (*13*) with pairwise comparison and maximum composite likelihood nucleotide substitution model. Phylogenetic trees were constructed by UPGMA (unweighted pair group method using arithmetic averages) with statistical significance of the phylogenetic analyses estimated by bootstrap analysis with 1,000 pseudoreplicate datasets. A and B represent phylogenetic trees of viral protein 1 sequences of enterovirus 71 and echovirus 13, respectively. The serotype, state and year of isolation of each strain and GenBank accession numbers of reference strains used are indicated. 1000B is an echovirus 1 strain. Scale bars indicate nucleotide substitutions per site.

**Figure 4:**
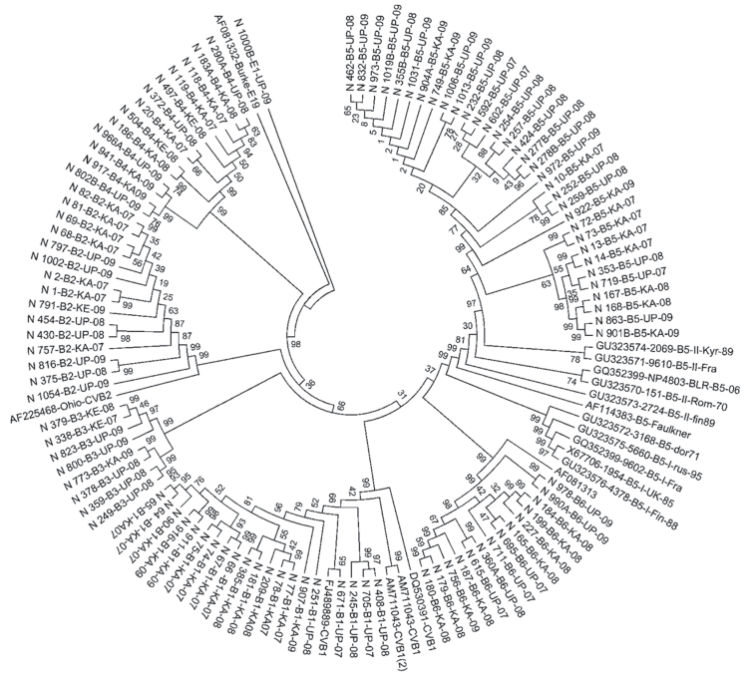
Phylogenetic tree of viral protein 1 sequences of coxsackievirus B1–B6 isolates generated in comparison with those of strains belonging to different genotypes of B1, B2, B3, B4, B5, and B6 serotypes, India, 2007–2009. Multiple sequence alignments were performed by using ClustalW program (www.genome.jp/tools/clustalw/) and phylogenetic analysis by MEGA5 program (*13*) with pairwise comparison and maximum composite likelihood nucleotide substitution model. Phylogenetic trees were constructed by UPGMA (unweighted pair group method using arithmetic averages) with statistical significance of the phylogenetic analyses estimated by bootstrap analysis with 1,000 pseudoreplicate datasets.

To understand the genetic relatedness of isolates in India that belonged to an EV type to those from other countries, we performed initially phylogenetic analyses of VP1 sequences of isolates from India that belonged to the prevalent EV71, E13, and CBV types with a large number of VP1 sequences representing different genogroups or subgenogroups within the types available in GenBank. Final phylogenetic trees, generated by using a few representative strains belonging to different genogroups and subgenogroups of EV71, E13, and CBV serotypes, are shown in Figure 3, panels A and B, and Figure 4. These studies showed that most of the India isolates segregated into either distinct genogroups or subgenogroups within known genogroups. Among the India EV71 isolates, 3 distinct new genogroups tentatively assigned as D (represented by N975c), E (N493), and F (N390), were identified (Figure 3, panel A). Isolate N975c showed ≈76%-nt sequence identity with a few EV71 strains but exhibited <70% aa identity, which suggests that it represents a new EV71 lineage. Other EV71 India strains segregated into different subgenogroups within C (C6 and C7) and B (B6) genogroups (*21*–*23*). Most India E13 strains formed 2 distinct subgenogroups within 2 E13 genogroups (Figure 3, panel B) (*24*–*26*). Most India CBV isolates, although grouped according to serotypes CBV1 to CBV6, represented a different subgenogroup within each type (Figure 4) (*27*,*28*). Preliminary phylogenetic analyses of VP1 sequences of strains belonging to a few other types also showed similar results (data not shown). These data suggest that most India EVs represent evolutionary lineages, within a given serotype, that differ from those prevalent in other countries.

## Discussion

This study, which represents a comprehensive molecular epidemiologic investigation of EVs associated with NP-AFP in India, was feasible because of the detection of a large number of AFP cases during the last several years from all regions of the country. The few previous studies from India and Pakistan identified 6–15 serotypes (*11*,*29*) in AFP cases by using methods that are limited in scope in identifying the >100 known HEV serotypes. Although studies in other countries (*14*,*15*,*30*,*31*) identified 6–12 serotypes, those in Romania and the People’s Republic of China observed 20 and 40, respectively (*32*,*33*). The serotypes that were prevalent varied in each of these studies. In contrast, we identified 66 NPEV serotypes in samples collected during 2007–2009 from children with NP-AFP in India. Detailed phylogenetic analysis of strains belonging to a few prevalent serotypes showed that NP-AFP is associated with circulation of strains belonging to different genogroups and subgenogroups of a serotype in widely separated geographic regions of the country (Figure 3, panels A and B; Figure 4).

Our results raise several issues that require immediate attention for the future direction of clinical and basic research and for better understanding of the health risks posed by NPEV infections. First, no virus was isolated in ≈70% of the AFP cases that remain to be identified. Second, the genetic nature of the viral agent(s) in ≈35% cases that were positive for virus in RD cells remains to be determined. Third, the association of a wide spectrum of serotypes with NP-AFP poses a challenging problem toward development of effective anti-enteroviral strategies. However, the need for effective antiviral strategies, including vaccines, is a major leap from the findings reported here and requires an in-depth analysis of the true effect of disease.

Detection of EV71 as the single most prevalent EV type associated with NP-AFP is of clinical significance because it is regarded as the most virulent neurotropic EV, next to poliovirus, associated with poliomyelitis-like paralytic disease, meningitis, meningoencephalomyelitis, Guillain-Barré syndrome, transeverse myelitis, cerebellar ataxia, opsoclonus-myoclonus syndrome, benign intracranial hypertension, brainstem encephalitis (*34*–*36*), and frequent epidemics of hand, foot, and mouth disease with substantial illness and death worldwide affecting >500,000 children in the Asia-Pacific region and causing >200 deaths in China during the past decade (*37*–*39*). The observation that >30% of EV71 detections involved mixed infections could be of clinical significance in terms of severity and spectrum of the diseases associated with these infections. Detection of 3 new genogroups of EV71 in widely separated geographic regions in India suggests uniform spread of EVs of different lineages of EV71 and other types in India. Long-term assessment of the clinical outcome of the EV71 infections would be of interest to better understand the severity of the disease in children with AFP.

Our study provides a wealth of information about NP-AFP in India. It suggests the necessity for the WHO-NPSP programs, which are primarily directed for a 60-day follow-up of poliomyelitis cases, to design and implement a long-term strategic plan for understanding and addressing the short-term and long term health risks in children arising from NPEV infections after poliomyelitis elimination in India.

Technical Appendix Primers for reverse transcription PCR; state and yearly distribution of enterovirus serotypes associated with acute flacid paralysis; enterovirus species, serotypes, and isolates associated with AFP; and enterovirus serotypes detected in mixed infections in children with acute flacid paralysis.
