# Supplementary material for: Antigenic Diversity of Enteroviruses Associated with Nonpolio Acute Flaccid Paralysis, India, 2007–2009
Source: Emerg Infect Dis. 2012 Nov;18(11):1833–40. doi: 10.3201/eid1811.111457 (PMC3559176; doi:10.3201/eid1811.111457)
Supplement: Technical Appendix — Primers for reverse transcription PCR; state and yearly distribution of enterovirus serotypes associated with acute flacid paralysis; enterovirus species, serotypes, and isolates associated with AFP; and enterovirus serotypes detected in mixed infections in children with acute flacid paralysis. [file 11-1457-Techapp-s1.pdf]

Article DOI: http://dx.doi.org/10.3201/eid1811.111457

Antigenic Diversity of Enteroviruses Associated with Nonpolio Acute Flaccid Paralysis, India, 2007–2009

Technical Appendix

This supplementary material provides additional information about the authors’ work on enteroviruses associated with nonpolio acute flaccid paralysis in India.

Primers for reverse transcription PCR

Oligonucleotides specific for enterovirus (EV) A, B, C, and D species, EV71, Aichivirus/Kobuvirus, Klassevirus, parechovirus, cardiovirus, and H-cosavirus were obtained from MWG Biotech (Bangalore, India). The primers contained degenerate nucleotides at specific positions. The picornavirus sequence corresponding to viral protein (VP)3 and 2A regions (VP1-specifc sequences in EV71 primers) located at the 5′ and 3′ ends, respectively of the VP1 gene is indicated in **boldface**. The primers contained sites for desired restriction endonucleases.

EV-B-F: 5′-TACACAAGCT TGAATTCGATATC **GYWTNGYDTC NGNTGCAAYG AYTTYTC**-3′ (2292-2318).

EV-B-R: 5′-CAATATCTCGAGAGATCTGGATC**CTGRCAYCTRGCDATDG TRTCRCA**-3′ (3501-3479).

EV-A-F: 5′-TACACAAGCTTGAATTCGATATC **CCHTGGATHA GYAACACWCA CT**-3′ (2258-2280).

EV-A-R: 5′-CAATATCTCGAGAGATCTGGATCC **GYTTDGAGAA RCTRACRGGG TAGT-**3′ (3644-3621).

EV-C-F:5′- TACACAAGCTTGAATTCGATATC **CACDATGGTD GYDCC WTGGAT**-3′ (2265-2286).

EV-C-R:5′- CAATATCTCGAGAGATCTGGATCC **GCTARRTGGT ARTTR MMRAT YTTGTA**-3′ (3586-3561).

EV-D-F: 5′- TACACAAGCTTGAATTCGATATC **AYGCYAARKC AAYMA ATGCTAAT**

GTT-3′ (2235-2260).

EV-D-R: 5′-CAATATCTCG AGAGATCTGG ATC**ACTAGRA CATCTGATTG CCAGTC**-3′ (3453-3431).

EV71-F: 5′-TACACAAGCT TGAATTCGAT ATC**GTCACHY TNGTNRTACC HTGGAT**-3′ (2207-2229).

EV71-F1: 5′-TACACAAGCT TGAATTCGAT **ATCTGGACAC TGGCAAAGTT CCAGCACT-**3′ (2555-2580).

EV71-R1: 5′-CAATATCTCG AGAGATCTGG ATCCA**AACTA GGTTTGCCCA ATCATTGT-**3′ (3427-3405).

Aichi-Kobu-F: 5′-TACACAAGCT TGAATTCGAT ATC**KSARCCH CGYACCACYT TC**-3′ (3076-3094).

Aichi-Kobu-R: 5′- CAATATCTCG AGAGATCTGG ATCC**TGTTGG GRAGKKVHWC RCCR GTGAT-**3′ (4266-4242).

Klassevirus-F: 5′-TACACAAGCT TGAATTCGAT ATC**CTTCTGA WKSTRWCYYC ACCACTG-**3′ (2874-2894).

Klassevirus-R: 5′-CAATATCTCG AGAGATCTGG ATCC**ATCCAR AGWTARTAGA RWGCAA-**3′ (4086-4065).

Parecho-F: 5′- TACACAAGCT TGAATTCGAT ATC**AATGCHA THTACACHAT HTGTGA**-3′(2086-2108)

Parecho-R: 5′- CAATATCTCG AGAGATCTGG ATCC**ATCCAC TCYTCMACMA YDAY CCARTCTG**-3′ (3221-3194)

Cardiovirus-VP1-F: 5′-TACACAAGCT TGAATTCGAT ATC**ACWCTT GGTTTCDGGH GG-**3′ (2696-2713).

Cardiovirus-VP1-R: 5′-CAATATCTCG AGAGATCTGG ATCC**TCGCCCA TRCASACRAG RA**-3′ (3739-3721).

H-Cosavirus-VP1-F: 5′-TACACAAGCT TGAATTCGAT ATC**CAYWCVN MKDBYDVHTT CTTCT-**3′ (2965-2986)

H-Cosavirus-VP1-R: 5′-CAATATCTCG AGAGATCTGG ATCC**TARTCNGGRWARCCATCAAA-3′** (3551-3532).

The primers were constituted into 4 sets. Set 1 contained EV-B and C-specific primers, set 2 contained those specific for EV-A, EV71 and EV-D and sets 3 and 4 consisted of primers specific for Aichi/Kobu and Klasseviruses, and cardio and H-cosaviruses, repectively.

Sequenced samples

Strains from independent nonpolio acute flaccid paralysis cases = 526

Additional strains from 41 mixed infections = 45

(37 cases with 2 strains and 4 with 3 strains)

Isolates from second specimens = 43

Strains from RD-negative samples = 4

Not sequenced = 6

———

Total 624

Table 1. State and yearly distribution of enterovirus serotypes associated with acute flaccid paralysis (includes isolates from mixed infections and second specimens)

		2007			2008			2009			
S no.	Serotype	Karnataka	Kerala	Uttar Pradesh	Karnataka	Kerala	Uttar Pradesh	Karnataka	Kerala	Uttar Pradesh	No.*
1	CV-A2	1	–	–	–	–	–	–	–	3	4
2	CV-A3		–	–	–	–	–	–	–	2	2
3	CV-A4	3	–	–	1	1	2	1	–	2	10 (3)
4	CV-A5	–	–	–	–	–		–	–	1	1
5	CV-A6	–	–	–	–	1	2	–	–	–	3
6	CV-A7	–	–	–	3	–	–	–	–	–	3 [1]
7	CV-A8	–	–	2	1	–	–	–	–	–	3 (1)
8	CV-A9	–	–	2	–	–	2	1	–	1	6
9	CV-A10	–	–	1	–	–	–	–	–	1	2
10	CV-A14	1	–	–	–	–	3	–	2	3	9
11	CV-A16	–	–		–	2	1	–	–	–	3
12	CV-A17	–	–	–	1	–	–	–	–	–	1
13	CV-A21	–	–	–	–	–	1	–	–	–	1
14	CV-B1	9	–	2	2	1	3	3	–	1	21 [4]
15	CV-B2	6	–	–	–	–	3	1	1	4	15 [3]
16	CV-B3	–	1	–	–	1	3	1	–	2	8
17	CV-B4	3	–	–	2	2	2	2	–	2	13 [1]
18	CV-B5	5	–	3	2	–	11	4	–	8	33 [4]
19	CV-B6	–	–	3	7	–	2	1	–	3	16 [1]
20	E-1	–	–	3	3	–	6	1	–	–	13 [1]
21	E-2	–	–	2	1	–	4	–	–	2	9
22	E-3	3	–	–	–	–	1	3	–	2	9 [1]
23	E-4	–	–	–	–	4	1	–	–	–	5
24	E-5	–	–	1	1	–	2	–	–	2	6
25	E-6	–	1	4	1	1	5	3	–	5	20
26	E-7	3	5	1	–	3	5	1	1	4	23 [1]
27	E-9	–	–	–	1	–	–	1	–	–	2
28	E-11	1	–	–	8	–	3	5	–	10	27 [1]
29	E-12	5	–	–	–	–	–	–	–	2	7 [2]
30	E-13	7	4	1	5	2	8	4	1	13	45 [4]
31	E-14	1	2	1	6	2	8	3	–	1	24 [4]
32	E-15	1	–	–	3	–	–	–	–	–	4 [1]
33	E-17	–	1	–	–	1	–	2	–	1	5
34	E-18	–		–	–	–	2	2	–	–	4
35	E-19	3	–	–	2	1	6	1	–	11	24 [1]
36	E-20	–	–	–	4	–	6	–	–	3	13 [1]
37	E-21	1	–	1	–	–	4	1	–	–	7
38	E-24	–	–	5	–	–	3	3	–	–	11 [1]
39	E-25	–	1	1	4	–	7	–	–	2	15
40	E-26	–	–	–	–	–	1	–	–	–	1
41	E-27	–	–	–	–	–	1	–	–	–	1
42	E-29	4	–	7	4	–	3	–	–	–	18 [2]
43	E-30	3	–	3	3	1	–	–	–	8	18 [2]
44	E-31	–	–	–	–	–	–	1	–	1	2
45	E-32	–	–	3	2	–	1	1	–	1	8
46	E-33	4	2	1	3	1	2	–	–	9	22 [3]
47	EV-69	–	–	–	1	–	4	1	–	6	12
48	EV-71	–	7	1	1	11	12	9	1	8	50 [1]
49	EV-73	–	–	–	1	–	1	–	–	–	2
50	EV-74	–	–	1	–	–	2	–	–	–	3
51	EV-75	1	–	5	1	–	2	1	1	2	13 [1]
52	EV-76	–	–	–	–	–	–	–	–	1	1
53	EV-78	–	–	–	–	–	3	–	–	1	4
54	EV-79	–	–	1	1	–	–	1	–	–	3
55	EV-80	2	–	1	1	–	–	–	1	–	5 [1]
56	EV-81	–	–	–	–	–	1	–	–	–	1
57	EV-83	–	–	2	–	–	3	–	–	1	6 [1]
58	EV-84	–	–	–	1	–	3	–	–	–	4
59	EV-86	–	–	–	1	–	–	–	–	–	1
60	EV-88	–	–	–	1	–	–	–	–	–	1
61	EV-89	–	–	–	–	–	1	–	–	–	1
62	EV-90	–	2	–	–	–		–	–	1	3
63	EV-93	–	–	–	–	–	3	–	–	1	4
64	EV-97	1	–	3	–	–	1	2	–	1	8
65	EV-100	–	–	2	–	–	–	–	–	2	4

S no.	Serotype	2007 Karnataka	2007 Kerala	2007 Uttar Pradesh	2008 Karnataka	2008 Kerala	2008 Uttar Pradesh	2009 Karnataka	2009 Kerala	2009 Uttar Pradesh	No.*
66	EV-107	–	–	–	–	–	–	–	–	1	1
Total		68	26	63	79	35	150	60	8	135	624

*Numbers in parentheses represent isolates identified from cell culture negative nonpolio acute febrile paralysis samples by nucleotide sequencing. Numbers in brackets represent isolates from second specimens RD-positive cases for which viral protein 1 sequences were determined.

Table 2. Enterovirus species, serotypes and isolates associated with acute flaccid paralysis for which nucleotide sequence and the nucleotide and amino acid identities among the isolates of each serotype were determined

S. no.	Serotype	Species	State KA	State UP	State KE	No. isolates	Identity, % nt	Identity, % aa	Isolates
1	CV-A2	HEV-A	1	3	–	4	80–81	94–95	N-115,845, 850A, 859
2	CV-A3	HEV-A	–	2	–	2	76	91	N-982, 984
3	CV-A4	HEV-A	5	4	1	10	85–87	89–97	**N-*48, 52, 56***, 183B, 253,357, 391, 928, 980,1062
4	CV-A5	HEV-A	–	1	–	1	73	90	N-864
5	CV-A6	HEV-A	–	2	1	3	78–82	87–96	N-258, 313, 384
6	CV-A7	HEV-A	3	–	–	3	81	93–95	N-132**, 133**, 183C
7	CV-A8	HEV-A	1	2	–	3	85	97–98	**N-*650***, 662, 672
8	CV-A9	HEV-B	1	5	–	6	78–79	91–95	N-451A,452,656,683,828,924
9	CV-A10	HEV-A	–	2	–	2	78–79	94–95	N-734, 992
10	CV-A14	HEV-A	1	6	2	9	80–84	89–97	N-92,470,471, 473, 510, 576, 994, 1047, 1055
11	CV-A16	HEV-A	–	1	2	3	74–76	87–90	N-442, 450B, 498
12	CV-A17	HEV-C	1	–	–	1	75	91	N-142
13	CV-A21	HEV-C	–	1	–	1	75	93	N-265A
14	CV-B1	HEV-B	14	6	1	21	72–98	88–96	N-64, **65**, 66, **67**, 74, **75**, 77, **78**, 90, 181, 209, 245, 251, 385, 408, 671, 705, 850B, 907, 911, 916
15	CV-B2	HEV-B	7	7	1	15	82–84	96–98	N-1, **2**, 68, **69**, 81, **82**, 375, 430, 454, 757, 791, 797, 816, 1002, 1054
16	CV-B3	HEV-B	1	5	2	8	78–79	95–97	N-249, 338, 359, 378, 379, 773, 800, 823
17	CV-B4	HEV-B	7	4	2	13	80–82	97–98	N-20, 118, **119**, 183A, 186, 290A, 372, 497, 504,802B, 917, 941, 966A
18	CV-B5	HEV-B	11	22	–	33	81–83	96–99	N-10, 13, **14**, 72, **73**, 167, **168**, 232, 252, 254, 257, 259, 277B, **278B**, 353, 355B, 424, 462, 592, 602,719, 749, 832, 863, 901B, 904A, 922, 972, 973, 1013, 1019B, 1031,1061
19	CV-B6	HEV-B	8	8	–	16	75–84	84–96	N-165, 179, **180**,184, 187, 199, 227, 360A, **361**, 615, 695, 711, 756, 978, 990A,1000B
20	E-1	HEV-B	4	9	–	13	75–82	92–97	N-136, 196, 272, 367, **368**, 400, 407, 482, 483, 658, 690, 733, 772
21	E-2	HEV-B	1	8	–	9	79–81	91–94	N- 220, 285, 406, 440, 441,668,742, 947, 1059A
22	E 3	HEV-B	6	3	–	9	79–81	94–97	N-116, 121, **122**, 405, 755, 763, 939, 1010, 1015
23	E-4	HEV-B	–	1	4	5	78–81	93–97	N-364, 510, 515, 517, 518
24	E-5	HEV-B	1	5	–	6	75–80	88–95	N-173, 468, 487, 713, 802A, 844
25	E-6	HEV-B	4	14	2	20	74–78	91–95	N-236, 284, 288, 333, 365, 394B,416, 587, 664, 700, 727, 909, 931, 932, 961,966B, 975A 961,985, 1007
26	E-7	HEV-B	4	10	9	23	75–84	87–94	N-15,**16**,89,240,276,290A,301,311,340,342,348, 352,381,392,394,572,699,750,783,956,957,959, 1021
27	E-9	HEV-B	2	–	–	2	78–79	89–97	N-460, 933
28	E-11	HEV-B	14	13	–	27	77–81	91–98	N-104, 194, 197, 202, 214, 216, 222, 228, **229**, 265B,281,409,908A,910,912,915,919,968,976,991,998A,999, 1009, 1028,1032,1046A,1064
29	E-12	HEV-B	5	2	–	7	78–82	96–98	N-79, **80**, 88, 100, **101**, 801, 1072
30	E-13	HEV-B	16	22	7	45	75–79	84–94	N-4,**5**,7,**8**,9,102,103,139,190,213,225,**226**,309,**310**, 316,327,331,349,355A,387,425,459,469,486,503,577,738,764,779,788,794,849,911A,914,953,976B,979,981,983,986,997,1003,1030, 1012,1074
31	E-14	HEV-B	10	10	4	24	75–80	83–95	N-91, 125, **126**, 129,**130**, 201, 218, 230, **231,** 279, **280**,320,323,325,337,413,437,500,575,613,925, 940,941A,1060
32	E-15	HEV-B	4	–	–	4	76–90	87–88	N-109, 131, 161, **162**
33	E-17	HEV-B	2	1	2	5	80–82	93–95	N-324, 382, 748, 936, 1017
34	E-18	HEV-B	2	2	–	4	78–80	93–95	N-242, 291, 766, 904B
35	E-19	HEV-B	6	17	1	24	77–78	88–99	N-

S. no.	Serotype	Species	State KA	State UP	State KE	No. isolates	Identity, % nt	Identity, % aa	Isolates
									86,**87**,96,182,205,261,417,426,429,431,432,513,744,795,949,950,964,975A,988,1004B,1008,1014,1025,1056
36	E-20	HEV-B	4	9	–	13	77–80	91–97	N-144, 176, 178, 263, 292, 297, 369, **370**, 411, 456, 948, 1046B, 1061
37	E-21	HEV-B	2	5	–	7	77–80	91–96	N-123, 410, 412, 422, 485, 726, 937
38	E-24	HEV-B	3	8	–	11	77–79	87–97	N- 282, **283**, 458, 661, 712, 714, 731, 736, 754, 935, 943
39	E-25	HEV-B	4	10	1	15	77–80	89–94	N-146, 156, 171, 273, 315,326, 356, 401, 404, 418, 438, 491, 706, 831, 862
40	E-26	HEV-B	–	1	–	1	78	88	268
41	E-27	HEV-B	–	1	–	1	77	88	256
42	E-29	HEV-B	8	10	–	18	78–80	93–96	N-70, **71**, 98, **99**, 153, 155, 193, 305, 398, 414, 435, 591, 655, 674, 678, 693A, 701, 728
43	E-30	HEV-B	6	11	1	18	75–81	88–98	N-85, 107, **108**, 148, 188, **189**, 505,584, 614, 665, 847, 944, 958, 995, 1000A, 1001, 1004A, 1019
44	E-31	HEV-B	1	1	–	2	80–81	96–98	N-761, 829
45	E-32	HEV-B	3	5	–	8	77–80	91–93	N-224, 443, 465, 722, 723, 737, 900, 990A
46	E-33	HEV-B	7	12	3	22	77–79	93–97	N-21,**22**,105,**106**,138,151,**152A**,329,346,419,420,519,730,873,977,998,1029A,1034,1058,1069,1059B,1071
47	EV-69	HEV-B	2	10	–	12	76–80	88–94	N-152B, 318, 434B, 439, , 453, 850C, 909A, 966C, 970, 974, 985A, 1050
48	EV-71	HEV-A	10	21	19	50	75–81	88–96	N-384,386,389,**390**,391B,427,444,445,446,447,449,450,451,471,474,475,480,490,493,494,498,502,505A,506,507,514,516,571,574,578,579,651,789,845,894,899,901A, 903, 905, 906, 910A, 918, 942, 955, 963, 975, 987, 993, 998, 1024
49	EV-73	HEV-B	1	1	–	2	75	88–96	N-150, 450A
50	EV-74	HEV-B	–	3	–	3	79	90–95	N-433,**434A**, 589
51	EV-75	HEV-B	3	9	1	13	84	92–100	N-83, 211, 247, **248,** 675, 684, 688, 694, 741, 784, 938, 951, 1043
52	EV-76	HEV-A	4	1	–	5			N-894, 895, 899, 905,1042
53	EV-78	HEV-B	–	4	–	4	79–83	94–97	N-303, 455, 457, 1039
54	EV-79	HEV-B	2	1	–	3	79–82	96–97	N-169, 696, 931
55	EV-80	HEV-B	3	1	1	5	75–79	89–96	N-94, **95**, 127, 660, 790
56	EV-81	HEV-B	–	1	–	1	79	95	N-428
57	EV-83	HEV-B	–	6	–	6	79–80	90–95	N-277, **278**, 489, 685, 698, 833
58	EV-84	HEV-B	1	3	–	4	81	96	N-246, 472, 476, 481
59	EV-86	HEV-B	1	–	–	1	89	98	N-160
60	EV-88	HEV-B	1	–	–	1	83	94	N-206
61	EV-89	HEV-A	–	1	–	1			N-286
62	EV-90	HEV-A	–	1	2	3			N-332, 341, 1052
63	EV-93	HEV-B	–	4	–	4	89–92	97–99	N-289, 423, 461, 1073
64	EV-97	HEV-B	3	5	–	8	90–97	93–98	N-6, 360B, 659, 693B, 729, 774, 804, 903
65	EV-100	HEV-B	–	4	–	4	82–85	93–96	N-653, 667, 821, 837
66	EV-107	HEV-B	–	1	–	1	81	93	N-842

KA, Karnataka; UP, Uttar Pradesh; KE, Kerala; HEV, human enterovirus. Independent strains from RD-positive cases 577, and isolates from second collection specimens 43 and strains from RD-negative cases 4. Numbers in **boldface** represent isolates from second specimens and those in ***bold italics*** represent isolates from cell culture–negative samples from children with nonpolio acute paralysis.

Table 3. Enterovirus serotypes detected in mixed infections in children with AFP*

1. N845-EV21 & N845A-EV71
2. N391-Cox A4 & N391A-EV71
3. N451A-CoxA9 & N451-EV71
4. N471-CoxA14 & N471A-EV71
5. N498A-CoxA16 & N498-EV71
6. N505- Ev30 & N505A- EV71
7. N901B-CoB5 & N901A-EV71
8. N998A-EV11 & N998B-EV71
9. N490B-EV30 & N490A-EV71
10. N903A-EV97 & N903- EV71
11. N384-A6 & N384A-EV71
12. N910-EV11 & N910A-EV71
13. N975A-EV6 & N975B-EV19 & N975C-EV71
14. N277B-CoxB5 & N277A-EV83
15. N355B-CoxB5 & N355A-EV13
16. N904A-CoxB5 & N904B-EV18
17. N1019B-CoxB5 & N1019A-EV30
18. N941-CoxB4 & N941A-EV14
29. N290A-CoxB4 & N290B-EV7
20. N802B-CoxB4 & N802A-EV5
21. N966A-CoxB4 & N966B-EV6 & N966C-EV69
22. N183B-CoxA4 & N183A-CoxB4 &N183C-CoxA7
23. N360A-CoxB6 & N360B-EV97
24. N990A-CoxB6 & N990B-EV32
25. N1000B-CoxB6 & N1000A-EV30
26. N911- CoxB1 & N911A-EV13
27. N850A-CoxA2 & N850-B-CoxB1 & N850C-EV69
28. N510- EV4 & N510A-CoxA14
39. N1059A-EV2 & N1059B-Ev33
30. N909- EV6 & N909A-EV69
31. N985- EV6 & N985A-EV69
32. N931-EV6 & N931A-EV69
33. N394B-EV6 & N394-EV7
34. N434A-EV9 & N434B-EV69
35. N1046A-EV11 & N1046B-Ev20
36. N976A-EV11 & N976B-EV13
37. N1004B-EV19 & N1004A-EV30
38. N693A-EV29 & N693B-EV97
49. N152A-EV33 & N152B-EV69
40. N450B-CoxA16 & N450A-EV73
41. N265A-CoxA21 & N265B-EV11

*AFP, acute flaccid paralysis.
